# Who Got Vaccinated for COVID-19? Evidence from Japan

**DOI:** 10.3390/vaccines9121505

**Published:** 2021-12-20

**Authors:** Toshihiro Okubo, Atsushi Inoue, Kozue Sekijima

**Affiliations:** 1 Faculty of Economics, Keio University, Tokyo 108-8345, Japan; 2 Nippon Institute for Research Advancement, Tokyo 150-6034, Japan; ainoue@nira.or.jp (A.I.); skozue@nira.or.jp (K.S.)

**Keywords:** COVID-19, vaccination behavior, socioeconomic factors, Japan

## Abstract

Vaccination has been critical to reducing infections and deaths during the coronavirus disease 2019 (COVID-19) pandemic. While previous studies have investigated attitudes toward taking a vaccine, studies on the determinants of COVID-19 vaccination behavior are scant. We examine what characteristics, including socioeconomic and non-economic factors, are associated with vaccination behavior for COVID-19 in Japan. We use a large nationwide online survey with approximately 10,000 participants. As of September 2021, 85% of the respondents said that they had received or would receive a COVID-19 vaccine. Employing logistic regression analysis on vaccination behavior, we found that vaccination rates are higher among those who are older, married, educated, and/or work in a large company. On the other hand, vaccination rates tend to be lower among the self-employed, younger women, and those with poor mental health. Income did not significantly correlate with vaccination. Medical workers were found to have a relatively high rate of vaccination. Although attitude towards risk and time preference were not crucial factors for vaccination, fear of infection, infection prevention behavior, and agreement with government policies on behavioral restrictions in crisis situations positively correlated with vaccination.

## 1. Introduction

Coronavirus disease 2019 (COVID-19) has spread around the world since January 2020. As of 10 December 2021, there were more than 267 million confirmed cases of infection and more than 5 million deaths due to COVID-19 worldwide [1]. In recent months, despite some waves of infection remaining, the spread of vaccines has attenuated the number of COVID-19-related deaths in many countries [2,3]. One of the keys to combating COVID-19 is a high rate of vaccination. Willingness toward vaccination is therefore important, and actually receiving a vaccine is crucial for the containment of COVID-19.

Most countries started public vaccinations in early 2021. The speed of vaccination has varied by country. As of April 2021, the proportion of people who had received at least one dose of an approved COVID-19 vaccine was 5% of the world population [4] (This is considered to be low to achieve herd immunity against COVID-19 [5]. However, the exact proportion of immune people needed to achieve herd immunity is difficult to estimate, as it depends on the type of virus, along with many other factors. Japan is a good example, being one of the countries which has a large gap between the proportion of people with immunity needed to achieve herd immunity and the proportion of people who are already immune [6]) (Note that the Johnson & Johnson vaccine, which had not been approved in Japan as of November 2021, requires only one dose). However, in some countries, full vaccination rates had drastically increased by summer or autumn 2021. Israel and the United Arab Emirates had the highest rates and speed of vaccination, with many other countries later hitting a ceiling. World governments adopted various policies and enacted various campaigns to promote vaccination.

In Japan, public vaccinations started with medical workers (about 4.7 million people) in February 2021, followed by people 65 years or older (about 36 million people) in April 2021. Vaccinations were then made available for all people 18 years or older in June 2021. Municipalities sent vouchers and vaccination-related notifications to their respective residents. Vaccine doses were administered by municipality offices, healthcare centers, and some hospitals under a reservation system accessible via a website and by phone. In addition, the Japanese government allowed some universities, large companies, and enterprises to provide vaccines. However, a wave of infections much larger than previous ones hit Japan in the period from July to September 2021. At its peak, on 20 August 2021, 25,992 new infections were confirmed. Amid increased public concern and fear, vaccination centers were overwhelmed with demand. Many people who reserved had to wait a few weeks to a few months for their vaccination. Getting vaccinated is not compulsory in Japan. It is done voluntarily, and vaccinations are not provided without the recipient’s consent. The cost of vaccination is fully covered by public funds, and thus there is no financial burden on those who wish to get vaccinated.

In the literature, many studies have investigated public hesitancy or willingness toward vaccination and found that it varies across individuals and countries [7,8,9]. For instance, a global survey of 19 countries found that vaccine acceptance has high heterogeneity [10]. Low COVID-19 vaccine acceptance rates were more pronounced in the Middle East, Eastern Europe, and Russia, whereas high rates were observed in East Asia and Southeast Asia [9].

Some studies have investigated the likelihood of vaccination based on attitudes toward it. According to many studies, women are less likely to accept a COVID-19 vaccine than men [9]. The association between age and willingness toward vaccination is not conclusive, although most studies show that older people are more willing to get vaccinated [11]. A systematic review of factors influencing vaccination behavior for the 2009 H1N1 influenza found that being older or a man tends to be a predictor of a greater likelihood to accept vaccination [12].

A number of other factors have been found in several studies. Influenza vaccination history is a strong predictor of vaccine acceptance [13]. Furthermore, protecting oneself as well as others has been shown to be the main reason for willingness to be vaccinated, and concerns about serious side-effects and the safety of vaccines have been shown to be the main reasons for unwillingness [14]. One study found that the reasons for public hesitancy include a general lack of trust and doubts about the efficacy of vaccines [15]. Individual preferences, such as risk preference [16], time preference [17], perceptions such as fear toward COVID-19 [18,19], knowledge regarding prevention, and control measures against COVID-19 [20] are also related to vaccination willingness.

Although there are previous studies on willingness to be vaccinated, it is relatively unknown who has actually received a vaccine. Some previous studies have explored the case of Japan, but their focus is on vaccine acceptance. By contrast, our paper uncovers vaccine uptake in Japan using a large nationwide survey, asking whether individuals actually received a vaccination or not. As far as we know, this paper is the first to investigate which factors crucially affect vaccine uptake. As with previous studies on vaccination willingness, we focus on many factors, including socioeconomic factors, non-economic factors (risk attitude and time preference), daily life under the pandemic (fear of infections and countermeasures against COVID-19), policy stance, and mental health. This rich set of variables makes the survey well suited to the present investigation. As shown in our estimation results, vaccine willingness or hesitancy varied depending on some, but not all, of these attributes.

Our estimation results indicated three main trends. First, people who are older, married, or educated tend to be more likely to accept the vaccine; however, income and gender were not significant. Second, people in specific occupations, such as healthcare, were more likely to have been vaccinated. Working for a large company (more than 500 employees) was also positively associated with accepting a vaccine. Third, fear of infection and preventive behavior were positively associated with vaccine acceptance; however, risk attitude and time preference were not significant.

## 2. Materials and Methods

### 2.1. Sample and Setting

We used a large-sample nationwide individual survey on COVID-19 conducted by Keio University and Nippon Institute for Research Advancement (NIRA) titled “Fifth Questionnaire Survey on the Effects of the Spread of COVID-19 on Telework-based Work Styles, Lifestyle, and Awareness” [21]. The survey conducted several issues such as telework use [22,23]. The sample comprised workers in Japan. Information regarding the sample was taken from people registered on a panel managed by Nikkei Research, Inc., Tokyo, Japan, an internet-based research company. While the survey was operated by Nikkei Research Inc., the web survey development was conducted by Relia, Inc., Tokyo, Japan, and the auxiliary work for the survey was conducted by Anet, Inc., Nagano, Japan. The survey was conducted in September 2021. We aimed to collect data on workers aged 15 and older from all regions of Japan.

To obtain a broadly representative sample of Japan’s working population, we conducted stratified random sampling. Specifically, in the first stage, Japan was stratified into 60 groups, according five regional classifications, six age groups, and two genders. The number of individuals in each group was distributed in accordance with basic resident register population ratios (Population Census 2019 by the Ministry of Internal Affairs and Communications). In the second stage, respondents were randomly selected and were sent an e-mail requesting their participation in the survey. The questionnaire posed questions about vaccination status, perceptions related to COVID-19 (the respondent’s policy stance and countermeasures against COVID-19), and various personal characteristics, including socioeconomic status (gender, age, income, education, occupation, and job status) and non-economic factors (such as risk attitude and time preference.

In the survey, the purpose of the study and our privacy policy were explained on the screen before the questionnaire began. Informed consent was obtained from all respondents, via agreement with the following statement: “This questionnaire includes questions on whether you have been vaccinated, whether you have been infected with COVID-19, and whether you have been affected by a natural disaster. The results of your responses will be processed statistically and will not be used to identify you personally. By understanding your situation accurately, we can propose a realistic plan for society to follow. We appreciate your understanding and cooperation. If you agree with the purpose of this survey, please answer “I agree”. If you choose “Disagree”, the questions will not be displayed”.

### 2.2. Data Collection

Nikkei Research, Inc. invited those registered on the company’s panel to participate in the survey via e-mail on 4 September 2021 and started collecting responses immediately. The survey was closed on 22 September 2021, when the quota of responses had been reached for all groups. In total, 10,644 responses were provided.

While the survey was taking place, the Japanese government declared a fourth state of emergency on 13 September 2021, for the Tokyo metropolitan area, the Osaka metropolitan area, and some other prefectures. Therefore, during part of the survey period, these prefectures were under a state of emergency.

### 2.3. Measures

The questions relevant to this study are listed in Table A1 and are explained below.

#### 2.3.1. Vaccination

**Figure 1:**
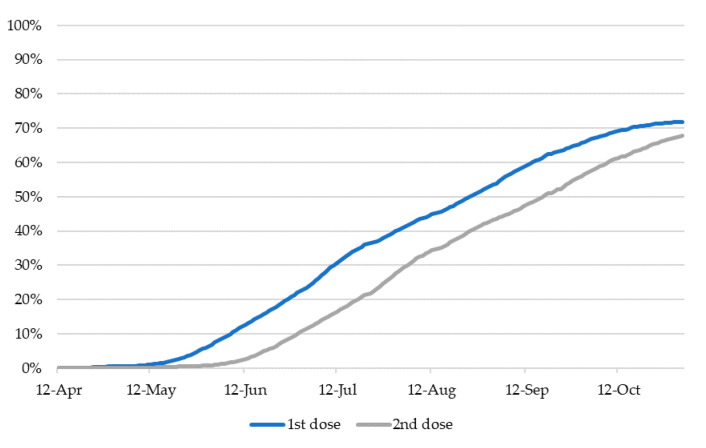
Vaccination rate in Japan.

Figure 1 shows the vaccination rates in Japan according to public vaccination data from the Cabinet Secretariat [24] (Figure 1 does not include the number of vaccinated medical personnel). As of 22 September 2021, when the survey was completed, 52% of the population in Japan had received their second vaccine dose, and 63% of the population had received their first. Therefore, the timing of the survey is suitable for investigating the factors influencing the decision to take the vaccine or not. As of 10 December 2021, 79% of the population had been fully vaccinated (first and second doses).

Our survey asked about vaccination behavior directly. Specifically, we offered the following multiple choice options to determine whether the participants had already been vaccinated against COVID-19: (1) “Received a second vaccine dose between February and June”; (2) “Received a second vaccine dose from July to September”; (3) “Received a first dose, but have not yet received a second”; (4) “Will receive a first dose in the future”; and (5) “Will not be vaccinated.” We categorized respondents who chose (1) to (4) as those who “have been vaccinated or will be vaccinated” and respondents who chose (5) as those who “will not be vaccinated”.

There is a rationale for categorizing respondents who chose (4) as those who “have been vaccinated or will be vaccinated”. Respondents who chose (4) accounted for 19.7% of our sample. Most of them were assumed to have already reserved their first vaccine dose or to be still searching for an available vaccination slot at a convenient time. In Japan, all residents received a coupon for vaccination issued by their municipalities. Vaccination is free. However, some individuals could not obtain their vaccination immediately, as it was necessary to first reserve a vaccination slot. In some cases, people had to wait weeks to months for a slot, because vaccination sites were limited to specific public places, such as city halls and health centers, and available only during working hours in their municipality of residence. Accordingly, the number of vaccinations per day was fairly limited. Furthermore, shortages in vaccine supply or vaccinators in some municipalities also slowed down the speed of rollout.

However, we cannot completely deny the possibility that a proportion of those who chose option (4) in fact “will not be vaccinated” (option (5)). Therefore, we also calculated the results if a proportion of (4) were categorized as (5) “will not be vaccinated”, as a robustness check. Nevertheless, all the main results held true, as discussed below.

#### 2.3.2. Socioeconomic Factors

To examine the relationship between socioeconomic factors and vaccination behavior, we measured individual characteristics such as gender, age, marital status, education, employment status, occupation, company size, prefecture of residence, and annual income in 2020. We used 38 occupational classifications, consisting of 12 major classification categories and 74 medium classification categories, taken from the Japanese Occupational Classification, as defined by the Ministry of Internal Affairs and Communications [25]. For the question on company size, the respondents were asked to select one of the following: (1) 1–4 people; (2) 5–29 people; (3) 30–99 people; (4) 100–499 people; (5) 500 people or more; (6) Public office).

#### 2.3.3. Risk Attitude and Time Preference

Regarding risk attitude, survey respondents were asked to read the following and choose one of 11 options from 0 to 10: “Are you generally a person who is fully prepared to take risks or do you try to avoid taking risks? Please choose one from ‘0 (not at all willing to take risks)’ to ‘10 (very willing to take risks)’”. This item is a Japanese translation of a question on general risk preferences used in the German Socio-Economic Panel (SOEP) survey [26].

We also asked about time preference, which is a measure of how much an individual might discount the future value or benefit of something when converting to its current value or benefit. A person with a low time preference is more self-controlled, while a person with a high time preference is more impulsive. Respondents were asked to read the following and choose an options from 1 to 8. “Instead of receiving 10,000 yen (about $88) after one month, what is the least amount you would be satisfied to receive after 13 months?” The options were as follows: 1 = 9500 yen (−5% annual interest rate); 2 = 10,000 yen (0% annual interest rate); 3 = 10,200 yen (2% annual interest rate); 4 = 10,400 yen (4% annual interest rate); 5 = 10,400 yen (6% annual interest rate); 6 = 11,000 yen (10% annual interest rate); 7 = 12,000 yen (20% annual interest rate); 8 = 14,000 yen (40% annual interest rate). This item is based on the question about time preference used in the “Japan Household Panel Survey on Consumer Preferences and Satisfaction” conducted by Osaka University in 2004.

#### 2.3.4. Perceptions of COVID-19

We measured fear related to COVID-19 with the question, “In the past 30 days, how often did you feel fear of COVID-19 infection?” Participants’ awareness of infection prevention was assessed with the following two questions: “In the past 30 days, how often did you pay attention to keeping physical distance (social distance)?” and “In the past 30 days, how often did you make a conscious effort to wear a mask outside the home?” Our estimation uses awareness of infection prevention by taking an average of the answers to the two question items.

#### 2.3.5. Policy Stance and Mental Health during the COVID-19 Pandemic

Variables expressive of individuals’ COVID-19 policy stance were also taken into account. One was whether they agreed or disagreed with restrictions on individual behavior imposed by the government in emergency situations. The other was whether they agreed or disagreed with policies that prioritized stimulating economic activity over deterring the spread of infection. Finally, we added the six-item Kessler Psychological Distress Scale (K6) to measure mental health. K6 is a scale proposed by Kessler et al. (2003) to measure and screen for mental illness [27]. The Japanese version, which we used in our survey, was developed by Furukawa et al. (2008) [28].

### 2.4. Statistical Analysis

Table 1 presents the descriptive statistics of the variables. It is followed by Table 2, which presents the result of the chi-square test, conducted to examine whether there were any significant differences in these variables between those classified as “have been vaccinated or will be vaccinated” and those classified as “will not be vaccinated.” Finally, the results of a logistic regression analysis on vaccination behavior are presented. The dependent variable was vaccination behavior (1 = vaccinated, 0 = non-vaccinated). Vaccination behavior was regressed on the above-mentioned socioeconomic characteristics (gender, age, income, education, employment status, occupation, firm size, and prefecture of residence), risk attitude, time preference, and perception of COVID-19 (countermeasures, policy attitude, and K6). In the estimation, we controlled for fixed effects resulting from the prefecture of residence. Table 2 also presents the adjusted odds ratios (ORs) and 95% confidence intervals (CIs). Of the 10,644 people who participated in the survey, 9304 (87%) were included in our analysis. Statistical analyses were performed using STATA version 15.0.

## 3. Results

### 3.1. Basic Statistics

Table 1 shows the basic statistics. The percentage of those who have been/will be vaccinated is 85%. Specifically, 65.6% of the respondents received at least a first dose, and 19.7% stated they will be vaccinated in the future. As discussed in Section 2.3.1, as of 22 September 2021, the completion date of the survey, the vaccination rate regarding at least the first dose was 63% in Japan as a whole, about the same as the rate in this data. It should be noted that the vaccination rates in our data are based on the population of workers in Japan and are not representative of Japan as a whole. The demographics of the sample were as follows: 15% were aged 15–29, 18% were aged 30–39, 24% were aged 40–49, 29% were aged 50–64, and 13% were aged 65 or above; 44% were female. About half the respondents in the sample were married, and about half held a college degree. Regarding employment status, more than 55% were regular employees, 31% were non-regular employees, and 11% were self-employed. According to the results of the 2015 census, the percentage of women in the Japanese workforce is 44%. The percentage of workers in each of the above age groups is 15%, 19%, 24%, 29%, and 13%, respectively. The percentage of workers who are also married is 63%, and 32% have graduated from university. In terms of employment status, 51% are regular employees, 28% are non-regular employees, and 12% are self-employed. The demographics of our sample are generally similar to those from the 2015 Census, although the percentage of college graduates in our sample was higher and that of married people was lower.

### 3.2. Chi-Square Test Result

Table 2 presents the results of the chi-square test. It shows that there were statistically significant differences between the vaccinated and non-vaccinated groups according to age, marital status, education, employment status, occupation, firm size, K6, perceptions of COVID-19, and policy stance regarding restrictions on individual behavior by the government in crisis situations. On the other hand, there were no significant differences between the two groups according to gender, annual income, risk preference, time preference, or policy stance for policies that prioritize stimulating economic activity over deterring the spread of infection.

### 3.3. Estimation Results

Table 3 presents the estimation results. The higher the age range, the larger the coefficients, from less than one to more than one in terms of odds ratio (OR). This indicates that as people get older, they were more likely to get vaccinated. In particular, people above age 65 were most likely to get vaccinated because they were a priority vaccination target. People who were married and those who were educated were more likely to be vaccinated. Self-employed people were less likely to be vaccinated than regular employees. Regarding occupation, public health nurses, medical technology and healthcare professionals, and professional social welfare workers (which includes people who work in facilities for the elderly and were therefore given priority for vaccination) had ORs significantly larger than one. This means that these medical/care workers were more likely to be vaccinated, probably because they were a priority target for vaccination. We note that being a doctor or a dentist was not significant in our results. This is because the occupational category we used includes not only medical doctors but also other types, such as veterinarians and pharmacists, who are not defined as healthcare professionals when it comes to COVID-19 vaccination priority. On the other hand, transport and post office clerical workers and office appliance operators were significantly less likely to get vaccinated (OR < 1). Regarding firm/company size, working for a company with more than 500 employees was found to be a significant predictor of vaccination. Employees of such companies are more likely to get vaccinated because group vaccination is provided by their employers.

Next, we turn to perception of COVID-19. Risk aversion and time preference were not found to be significant, whereas fear of COVID-19 and countermeasures were (OR > 1). Rather than risk attitude and time preference, individuals’ fear and preventive behavior crucially affected vaccination behavior.

Turning to policy stance, those who agreed with government restrictions in crisis situations tended to get vaccinated. On the other hand, whether people agreed with the government prioritizing economic policy over infection controls was not significant. This means that even if people prefer financial support to infection controls, they tend to get vaccinated, suggesting that they may believe that vaccination is effective as an economic measure through infection control. Respondents’ preference for policies that prioritize economic support or infection controls during the pandemic was not found to be a crucial factor for vaccination. 

Finally, mental health condition, measured by K6, showed an OR significantly smaller than 1. We note than if the variable score is 1, the respondent’s mental condition is poor. Thus, this result indicates that people with good mental health were more likely to be vaccinated. If someone is suffering from poor mental health, they may be hesitant toward vaccination, or their depression may prevent them from making a reservation for vaccination.

### 3.4. Additional Results

Table 4 reports the same estimation as Table 3 but with the sample split by gender. The results are almost the same as in Table 3; however, one contrast can be seen in the age categories. Being a younger woman is significant (OR < 1), whereas being a man in the same age range was not. The magnitude of coefficients was smaller than for men, indicating that younger women were more likely to be hesitant toward getting vaccinated.

Additionally, we conducted the same estimation using another definition of vaccination as robustness check. As mentioned above, those who chose (4) “Will receive a first dose in the future” can be categorized as unvaccinated. In this setting, estimation results were similar to Table 3 and Table 4. Due to the limited space, we omit this table from the present report, but can provide the results upon request.

## 4. Discussion

There have been several previous studies on vaccine hesitancy in Japan. One found that 47% of respondents said they were willing to receive a vaccine, 22% said they were not, and another 31% were indecisive [29]. Similarly, a study using a large-scale survey found that the percentages of respondents who answered not willing and not sure regarding intention to be vaccinated were 11.0% and 32.9%, respectively [30]. Accordingly, it can be said that about half of the Japanese population intend to receive a vaccine. In addition, various factors that are positively associated with willingness to receive a COVID-19 vaccine have been reported. In terms of socioeconomic factors, these are: male gender [29,30,31,32], older age [29,30,31,32], having children [29], high income [29,31], and living in a rural area [32]. The health factors are having underlying health problems [30,31] and good subjective health [29]. The psychological factors are fear of infection [30,31], concerns about the side-effects and safety of the vaccine [30], doubts about the effectiveness of the vaccines [31], willingness to vaccinate oneself to protect others from infection [31], trust in scientists, public authorities, and media [30], and general anxiety about the future [29].

In contrast to previous studies on vaccine acceptance and hesitancy, our focus is to investigate who actually received and who rejected vaccination. Willingness to accept vaccination would affect vaccine-receiving behavior. However, vaccine acceptance is not identical to vaccine-receiving behavior. While around 30% of the Japanese are unsure or neutral regarding vaccine acceptance, as mentioned above, the government’s vaccine campaign sought to promote vaccination for all people. As of 10 December 2021, 79% of the population had been vaccinated (at least the first dose). Cases of allergic reactions to vaccines were reported in the media and, as a result, people wanting to avoid potential side-effects may have decided against receiving a vaccine. On the other hand, the number of COVID-19-related deaths drastically increased in Japan from July to September 2021 and people closely related to each other were more likely to be infected, which might have increased fear of infections and led to people taking more countermeasures. In such circumstances, people who were initially hesitant to receive a vaccine might have decided to receive it.

In more detail, as shown in our estimation results, vaccine uptake behavior is different from willingness or hesitancy toward vaccination, which have been investigated in several previous studies [7,8,9]. There are several reasons for this discrepancy.

First, our result is largely affected by the public vaccination scheme. Vaccination in Japan is free and thus all people can receive it. As a result, income is not crucial factor. By contrast, as shown in previous studies [31,33,34], willingness toward vaccination is positively correlated with income. This indicates that if we ask only about intention toward vaccination without information on vaccination being free, people may consider the cost, and lower-income individuals may be more hesitant toward vaccination. Since the government provides free vaccination, income is not crucially related to the people who received a vaccine.

Second, the Japanese government allowed some large companies, universities, and public or semi-public bodies to provide vaccinations. Thus, many employees of large companies have easy access to vaccination at their office. This is why employees in large companies are positively associated with vaccination behavior. Furthermore, the government initially prioritized vaccination for healthcare workers in February 2021, followed by older people (65 or older) in April 2021, with vaccination for all other people starting from June 2021. The priority vaccination targeting older people and medical workers may be one cause of their positive correlation with vaccine-receiving behavior; however, it might also be because older people are susceptible to COVID-19 and medical workers face higher risk of infection, and thus both groups are more likely to get vaccinated.

Third, time preference and risk attitude were found not to crucially affect vaccination intention. This could be a result of a trade-off of vaccination benefits and side-effects. People benefit from vaccinations, yet they might have side-effects. Thus, the discounted future benefits could dissuade some people from getting vaccinated. Risk attitude might be also important. Risk-averse people would weigh the risk of infection without vaccination against the side-effects of vaccines. Therefore, time preference and risk attitude are positively or negatively correlated with vaccination, leading to a lack of significance in our results.

Lastly, the fourth wave of infections hit Japan in summer 2021, with record high numbers of new daily cases. Many infected people could not be hospitalized in some major cities because hospitals were filled to capacity. People thus experienced substantially increased fear of infection. In contrast to individual risk attitude and time preference, people gave higher priority to infection prevention. This might explain why fear of infection and willingness to take countermeasures in daily life were positively associated with vaccination, whereas risk and time preference were not significant factors.

During the COVID-19 pandemic, Japanese people tended to respond positively to the government’s policies. Overall, the government vaccination policies such as office vaccinations and priority targets for older people and medical workers worked well and resulted in a high vaccination rate. This might be largely dependent on characteristics of the population. The Japanese tend to put the interests of society over the interests of individuals, which affects vaccination behavior.

Finally, we admit there are some data qualifications. First, the survey sample consists of Japanese workers rather than the general population. It includes aging workers, but since retirement age is 65 to 70 in Japan, the age group above 65 is relatively small. According to the Labor Force Survey (Ministry of Internal Affairs and Communications), the percentage of working people in the population aged 40–49 accounts for 79%, while the percentage above 65 is only 25%. This might have led to bias in our estimation results, particularly in the age category. Second, the survey was not exhaustive but employed stratified random sampling, as mentioned in Section 2.1.

## 5. Conclusions

This paper investigated which characteristics (socioeconomic and non-economic factors) affect individuals’ COVID-19 vaccination behavior in Japan, by means of a large nationwide survey. While previous studies have investigated willingness or hesitancy toward vaccination, our focus was on vaccination behavior, that is, whether such individuals ultimately received a vaccine and were fully vaccinated or not. As of September 2021, the percentage of participants who responded that they had received or planned to receive a COVID-19 vaccine was 85%. As a result of the estimation process, we found that older people, married people, educated people, and workers in large companies were more likely to get vaccinated. On the other hand, self-employed persons, younger women, and people with poor mental health tended to be less likely to get vaccinated. Income did not significantly correlate with vaccination. Medical workers tended to be vaccinated, probably because they face a high risk of infection and were targeted by prioritized vaccination. Although risk attitude and time preference were not found to be crucial factors for vaccination, fear of infection and countermeasures against COVID-19, and agreement with government regulations in crisis situations were positively correlated with vaccination. Future research will investigate the relationship between vaccination hesitancy and vaccination uptake behavior.

## Figures and Tables

**Table 1:** Basic Statistics.

		Mean	SD	Min	Max
Vaccinated		0.85	0.35	0	1
Female		0.44	0.50	0	1
Age					
	15–29	0.15	0.36	0	1
	30–39	0.18	0.38	0	1
	40–49	0.24	0.43	0	1
	50–64	0.29	0.46	0	1
	>65	0.13	0.34	0	1
Married		0.51	0.50	0	1
College educated		0.51	0.50	0	1
Employment Status					
	Regular	0.55	0.50	0	1
	Non-regular	0.31	0.46	0	1
	Directors	0.02	0.15	0	1
	Self-employed	0.11	0.31	0	1
	Others	0.01	0.09	0	1
Income (million yen)		4.36	3.75	0.25	21.25
Occupation					
	Administrative and managerial workers	0.09	0.29	0	1
	Researchers	0.01	0.11	0	1
	Agriculture, forestry, and fishery engineers	0.00	0.06	0	1
	Manufacturing engineers	0.04	0.20	0	1
	Architects, civil engineers and surveyor	0.02	0.15	0	1
	Data processing and communication engineers	0.04	0.19	0	1
	Doctors, dentists, veterinarians, and pharmacists	0.01	0.12	0	1
	Public health nurses, midwives, and nurses	0.02	0.13	0	1
	Medical technology and healthcare professionals	0.02	0.13	0	1
	Professional social welfare workers	0.02	0.12	0	1
	Legal professionals	0.00	0.06	0	1
	Management, finance and insurance professionals	0.01	0.08	0	1
	Management and business consultants	0.00	0.06	0	1
	Teachers	0.03	0.16	0	1
	Authors, journalists, editors	0.00	0.06	0	1
	Artists, designers, photographers, film operators	0.01	0.11	0	1
	Other specialist professionals	0.01	0.11	0	1
	General clerical workers	0.17	0.38	0	1
	Accountancy clerks	0.03	0.17	0	1
	Production-related clerical workers	0.01	0.10	0	1
	Sales clerks	0.05	0.21	0	1
	Outdoor service workers	0.00	0.03	0	1
	Transport and post clerical workers	0.01	0.10	0	1
	Office appliance operators	0.00	0.05	0	1
	Sales workers	0.07	0.26	0	1
	Workers in family life support and care service	0.01	0.12	0	1
	Occupational health and hygiene service workers	0.01	0.09	0	1
	Food and drink cooking, staff serving customers	0.03	0.18	0	1
	Manager of residential facilities and buildings	0.01	0.09	0	1
	Other service workers	0.06	0.24	0	1
	Security workers	0.01	0.10	0	1
	Agriculture, forestry and fishery workers	0.00	0.07	0	1
	Manufacturing process workers	0.04	0.19	0	1
	Transport and machine operation workers	0.01	0.09	0	1
	Construction and mining workers	0.01	0.08	0	1
	Carrying, cleaning, packaging, and related workers	0.02	0.15	0	1
	Other	0.09	0.29	0	1
Enterprise size					
	1–4	0.14	0.35	0	1
	5–29	0.17	0.37	0	1
	30–99	0.17	0.37	0	1
	100–499	0.19	0.39	0	1
	More than 500	0.28	0.45	0	1
	Government offices	0.05	0.22	0	1
Risk aversion		3.93	2.25	0	10
Time preference		6.17	2.03	1	8
Perceived fear of COVID-19 infection		2.56	1.27	1	5
COVID-19 preventive behaviors		3.32	1.33	1	5
Agree with the restrictions on individual behavior by the government in emergency situations		0.44	0.94	−2	2
Agree with the policies that prioritize stimulating economic activity over deterring the spread of infection		0.22	0.96	−2	2
K6 over 5 (possibility of having some depression/anxiety issues)		0.39	0.49	0	1

Note: N = 9304.

**Table 2:** Chi-square test.

		Vaccination				
		Vaccinated		Non-Vaccinated		
		n	%	n	%	*p*-Value
Gender						0.249
	Female	3479	43.81	620	45.49	
	Male	4462	56.19	743	54.51	
Age						<0.001
	15–29	1076	13.55	316	23.18	
	30–39	1341	16.89	334	24.50	
	40–49	1919	24.17	359	26.34	
	50–64	2433	30.64	292	21.42	
	>65	1172	14.76	62	4.55	
Marital status						<0.001
	Unmarried	3745	47.16	851	62.44	
	Married	4196	52.84	512	37.56	
Education						<0.001
	Not college educated	3777	47.56	752	55.17	
	College educated	4164	52.44	611	44.83	
Employment Status						<0.001
	Regular	4374	55.08	723	53.04	
	Non-regular	2491	31.37	421	30.89	
	Directors	204	2.57	22	1.61	
	Self-employed	809	10.19	176	12.91	
	Others	63	0.79	21	1.54	
Occupation						<0.001
	Administrative and managerial workers	780	9.82	91	6.68	
	Researchers	91	1.15	14	1.03	
	Agriculture, forestry, and fishery engineers	25	0.31	5	0.37	
	Manufacturing engineers	312	3.93	58	4.26	
	Architects, civil engineers and surveyor	197	2.48	27	1.98	
	Data processing and communication engineers	318	4.00	47	3.45	
	Doctors, dentists, veterinarians, and pharmacists	115	1.45	14	1.03	
	Public health nurses, midwives, and nurses	136	1.71	12	0.88	
	Medical technology and healthcare professionals	155	1.95	10	0.73	
	Professional social welfare workers	128	1.61	13	0.95	
	Legal professionals	29	0.37	7	0.51	
	Management, finance and insurance professionals	54	0.68	7	0.51	
	Management and business consultants	33	0.42	3	0.22	
	Teachers	224	2.82	25	1.83	
	Authors, journalists, editors	28	0.35	5	0.37	
	Artists, designers, photographers, film operators	95	1.20	23	1.69	
	Other specialist professionals	107	1.35	15	1.10	
	General clerical workers	1412	17.78	208.00	15.26	
	Accountancy clerks	246	3.10	31	2.27	
	Production-related clerical workers	90	1.13	9	0.66	
	Sales clerks	383	4.82	68	4.99	
	Outdoor service workers	8	0.10	1	0.07	
	Transport and post clerical workers	68	0.86	23	1.69	
	Office appliance operators	20	0.25	6	0.44	
	Sales workers	552	6.95	118	8.66	
	Workers in family life support and care service	119	1.50	9	0.66	
	Occupational health and hygiene service workers	68	0.86	12	0.88	
	Food and drink cooking, staff serving customers	263	3.31	51	3.74	
	Manager of residential facilities and buildings	65	0.82	3	0.22	
	Other service workers	474	5.97	92	6.75	
	Security workers	80	1.01	12	0.88	
	Agriculture, forestry and fishery workers	38	0.48	5	0.37	
	Manufacturing process workers	277	3.49	70	5.14	
	Transport and machine operation workers	63	0.79	16	1.17	
	Construction and mining workers	42	0.53	11	0.81	
	Carrying, cleaning, packaging, and related workers	184	2.32	39	2.86	
	Other	662	8.34	203	14.89	
Enterprise size						0.008
	1–4	1095	13.79	224	16.4	
	5–29	1303	16.41	246	18.1	
	30–99	1322	16.65	230	16.9	
	100–499	1503	18.93	262	19.2	
	More than 500	2301	28.98	339	24.9	
	Government offices	417	5.25	62	4.6	
K6						<0.001
	Less than 5	4900	61.71	772	56.64	
	Over 5 (possibility of having some depression/anxiety issues)	3041	38.29	591	43.36	
		Mean	SD	Mean	SD	
Income (million yen)		4.39	0.04	4.16	0.11	0.033
Risk aversion		3.92	0.03	3.94	0.06	0.760
Time preference		6.18	0.02	6.14	0.06	0.498
Perceived fear of COVID-19 infection		2.60	0.01	2.29	0.04	<0.001
COVID-19 preventive behaviors		3.40	0.01	2.90	0.04	<0.001
Agree with the restrictions on individual behavior by the government in emergency situations		0.48	0.01	0.22	0.03	<0.001
Agree with the policies that prioritize stimulating economic activity over deterring the spread of infection		0.22	0.01	0.22	0.02	0.819

**Table 3:** Basic Estimation Results.

		OR	95% CI		*p*-Value
Female		0.89	0.77	1.02	0.086
Age	15–29	0.75	0.65	0.86	<0.001
	30–39	0.76	0.63	0.90	0.002
	40–49	Ref			
	50–64	1.62	1.32	1.97	<0.001
	>65	3.98	2.75	5.77	<0.001
Married		1.36	1.15	1.61	<0.001
College educated		1.30	1.11	1.53	0.001
Employment Status	Regular	Ref			
	Non-regular	0.89	0.76	1.05	0.181
	Directors	1.07	0.69	1.68	0.753
	Self-employed	0.65	0.49	0.87	0.004
	Others	0.67	0.41	1.10	0.113
Income		1.00	0.98	1.02	0.676
Occupation	Administrative and managerial workers	Ref			
	Researchers	1.03	0.58	1.82	0.916
	Agriculture, forestry, and fishery engineers	1.44	0.51	4.03	0.493
	Manufacturing engineers	0.98	0.72	1.32	0.867
	Architects, civil engineers and surveyor	1.28	0.84	1.94	0.254
	Data processing and communication engineers	1.25	0.91	1.73	0.166
	Doctors, dentists, veterinarians, and pharmacists	1.13	0.67	1.92	0.652
	Public health nurses, midwives, and nurses	2.36	1.44	3.88	0.001
	Medical technology and healthcare professionals	3.11	1.62	5.99	0.001
	Professional social welfare workers	1.90	1.02	3.54	0.044
	Legal professionals	0.80	0.41	1.53	0.495
	Management, finance and insurance professionals	1.27	0.51	3.18	0.613
	Management and business consultants	1.81	0.50	6.49	0.366
	Teachers	1.27	0.87	1.86	0.220
	Authors, journalists, editors	0.89	0.39	2.04	0.775
	Artists, designers, photographers, film operators	0.96	0.66	1.40	0.833
	Other specialist professionals	1.21	0.60	2.46	0.592
	General clerical workers	1.25	1.01	1.53	0.037
	Accountancy clerks	1.30	0.87	1.93	0.198
	Production-related clerical workers	1.69	0.91	3.13	0.099
	Sales clerks	0.90	0.61	1.32	0.583
	Outdoor service workers	1.66	0.20	13.61	0.636
	Transport and post clerical workers	0.53	0.35	0.81	0.003
	Office appliance operators	0.42	0.20	0.88	0.022
	Sales workers	0.88	0.67	1.16	0.373
	Workers in family life support and care service	2.10	0.95	4.65	0.068
	Occupational health and hygiene service workers	1.07	0.56	2.05	0.830
	Food and drink cooking, staff serving customers	1.12	0.76	1.66	0.563
	Manager of residential facilities and buildings	2.07	0.67	6.41	0.206
	Other service workers	0.97	0.68	1.38	0.849
	Security workers	1.01	0.45	2.24	0.984
	Agriculture, forestry and fishery workers	1.52	0.57	4.09	0.406
	Manufacturing process workers	0.76	0.51	1.12	0.168
	Transport and machine operation workers	0.81	0.42	1.57	0.533
	Construction and mining workers	0.72	0.30	1.71	0.452
	Carrying, cleaning, packaging, and related workers	0.93	0.58	1.47	0.747
	Other	0.72	0.53	0.97	0.031
Enterprise size	1–4	Ref			
	5–29	1.22	0.95	1.56	0.124
	30–99	1.33	1.04	1.70	0.024
	100–499	1.33	0.98	1.81	0.064
	More than 500	1.46	1.13	1.88	0.004
	Government offices	1.42	1.02	1.96	0.036
Risk aversion		1.01	0.98	1.04	0.519
Time preference		0.99	0.96	1.02	0.412
Perceived fear of COVID-19 infection		1.16	1.09	1.23	<0.001
COVID-19 preventive behaviors		1.22	1.14	1.30	<0.001
Agree with the restrictions on individual behavior by the government in emergency situations		1.25	1.17	1.34	<0.001
Agree with the policies that prioritize stimulating economic activity over deterring the spread of infection		0.96	0.89	1.04	0.315
K6 over 5 (possibility of having some depression/anxiety issues)		0.73	0.63	0.85	<0.001
Control	Prefecture	✓			
N		9304			
Log likelihood		−3507.0			

**Table 4:** Estimation results by gender.

		Male				Female			
		(1)				(2)			
		OR	96% CI		*p*-Value	OR	97% CI		*p*-Value
Age	15–29	0.878	0.691	1.116	0.288	0.578	0.438	0.762	<0.001
	30–39	0.914	0.680	1.229	0.553	0.576	0.443	0.749	<0.001
	40–49	Ref				Ref			
	50–64	1.669	1.266	2.200	<0.001	1.499	1.188	1.893	0.001
	>65	4.816	2.997	7.739	<0.001	3.139	1.967	5.010	<0.001
Married		1.463	1.143	1.871	0.002	1.253	1.033	1.521	0.022
College educated		1.301	1.055	1.604	0.014	1.348	1.069	1.699	0.012
Employment Status	Regular	Ref				Ref			
	Non-regular	0.888	0.710	1.110	0.297	0.847	0.666	1.077	0.175
	Directors	1.477	0.782	2.788	0.229	0.575	0.254	1.299	0.183
	Self-employed	0.688	0.413	1.148	0.152	0.569	0.379	0.856	0.007
	Others	0.621	0.308	1.252	0.183	0.714	0.381	1.339	0.294
Income		0.985	0.953	1.018	0.365	1.008	0.970	1.047	0.688
Enterprise size	1–4	Ref				Ref			
	5–29	1.140	0.735	1.770	0.558	1.259	0.839	1.888	0.266
	30–99	1.259	0.806	1.966	0.312	1.425	0.941	2.156	0.094
	100–499	1.256	0.781	2.021	0.347	1.435	0.997	2.064	0.052
	More than 500	1.382	0.899	2.123	0.140	1.572	1.095	2.258	0.014
	Government offices	1.460	0.883	2.414	0.140	1.351	0.827	2.208	0.230
Risk aversion		1.005	0.976	1.035	0.724	1.016	0.976	1.058	0.438
Time preference		1.005	0.971	1.041	0.771	0.970	0.926	1.016	0.199
Perceived fear of COVID-19 infection		1.133	1.044	1.230	0.003	1.195	1.060	1.347	0.004
COVID-19 preventive behaviors		1.271	1.165	1.386	<0.001	1.151	1.048	1.265	0.003
Agree with the restrictions on individual behavior by the government in emergency situations		1.293	1.191	1.402	<0.001	1.177	1.065	1.301	0.001
Agree with the policies that prioritize stimulating economic activity over deterring the spread of infection		0.944	0.849	1.050	0.291	0.972	0.872	1.085	0.616
K6 over 5 (possibility of having some depression/anxiety issues)		0.671	0.529	0.851	0.001	0.795	0.639	0.990	0.041
Control	Occupation	✓				✓			
	Prefecture	✓				✓			
N		5197				4095			
Log likelihood		−1881.5				−1573.3			

## Data Availability

The data for our paper are available from the corresponding author on reasonable request. The data are not publicly available due to privacy concerns.

## Appendix A

**Table A1:** Definition of variables.

Variable	Explanation of Variable
Vaccinated	“Have you received the vaccine against COVID-19?” (1 = Received the second dose of vaccination between February and June; Received the second dose of vaccination from July to September; Received the first dose, but have not yet received the second one; Will receive the first dose in the future; 0 = Will not be vaccinated.)
Socioeconomic Factors	
Gender	“What is your gender?”
Age	“What is your age?”
Marital status	“Please indicate who you are currently living with.” We classified individuals as “married” if they answered “Spouse (including de facto marriage),” and “not married” if not.
Education	“What is your last educational background (including current and correspondence courses)?” We classified individuals as “college educated” if they answered “university undergraduate,” “master’s program, professional graduate school,” or “postdoctoral program,” and “not college educated” if they answered others.
Employment status	“What is your employment status?” We classified individuals as “regular” if they answered “employee (regular employee),” “non-regular” if they answered “employee (part-time worker, dispatched worker, contract employee, commissioned worker, and others),” “director” if they answered “director of a company, etc.," “self-employed” if they answered “self-employed (with employees),” “self-employed (with no employees),” or “self-employed helper,” and “others” if they answered “housewife/househusband,” “student,” “unemployed,” or “others.”
Occupation	“What is your occupation?”
Enterprise size	“Which of the following is the number of employees (including part-time workers and dispatched workers, etc.) in your company or business as a whole? If you work for a public office, please select “Public office.”
Prefecture	“Please indicate the prefecture in which you live.”
Income	“How much did you personally earn from your main job in 2020? Please indicate the amount before taxes and insurance premiums are deducted. If you are self-employed, please indicate the amount of operating income after subtracting necessary expenses from net sales.”
Personal Preference	
Risk preference	“Are you generally a person who is fully prepared to take risks or do you try to avoid taking risks?” (0 = “not at all willing to take risks” to 10 = “very willing to take risks.”)
Time preference	“Instead of receiving 10,000 yen (approximately $88) after one month, how much would you be satisfied with receiving at least after 13 months?” (1 = “9500 yen (-5% annual interest rate),” 2 = “10,000 yen (0% annual interest rate),” 3 = “10,200 yen (2% annual interest rate),” 4 = “10,400 yen (4% annual interest rate),” 5 = “10,600 yen (6% annual interest rate),” 6 = “11,000 yen (10% annual interest rate),” 7 = “12,000 yen (20% annual interest rate),” 8 = “14,000 yen (40% annual interest rate).”)
Perceptions of the COVID-19	
Perceived fear of COVID-19 infection	“In the past 30 days, how often did you feel fear of the COVID-19 infection?” (1 = Always to 5 = Not at all).
COVID-19 preventive behaviors	“In the past 30 days, how often did you pay attention to keeping physical distance (social distance)?” (1 = Always to 5 = Not at all.) and “In the past 30 days, how often did you make a conscious effort to wear a mask outside the house?” (1 = Always to 5 = Not at all.) We measure the average perception of infection prevention by adding the results of the two item responses and dividing by two.
Attitudes toward the policy	
Agree with the restrictions on individual behavior by the government in crisis situations	“We would like to ask you a question in light of the spread of the COVID-19. Do you agree or disagree that the government should take the following measures for the entire nation, including the future?“—“Restrictions on individual behavior and control of goods and economy by the government in emergency situations.” (−2 =“Disagree,” −1 = “Somewhat disagree,” 0 = “Neither agree nor disagree/Don’t know,” 1 = “Somewhat agree,” 2 = “Agree.”)
Agree with the policies that prioritize stimulating economic activity over deterring the spread of infection	Same question as above—“Promote policies that prioritize stimulating economic activity over deterring the spread of infection.” (−2 = “Disagree,” −1 = “Somewhat disagree,” 0 = “Neither agree nor disagree/Don’t know,” 1 = “Somewhat agree,” 2 = “Agree.”)
Mental health	
Kessler-6 Non-Specific Psychological Distress Scale (K6)	“In the past 30 days, how often did you feel: (1) so sad nothing could cheer you up?; (2) nervous?; (3) restless or fidgety?; (4) hopeless?; (5) that everything was an effort?; (6) worthless? ”(0 = Not at all to 4 = Always.) We added up the scores and created a dummy variable with 1 for those who scored 5 or more, indicating the possibility of having some depression/anxiety issues, and 0 for those who scored less than 5.
